# Exploring the Self-Reported Physical and Psychological Effects in a Population Exposed to a Regional Conflict

**DOI:** 10.1007/s10900-024-01337-6

**Published:** 2024-02-23

**Authors:** Naama Shamir-Stein, Ilana Feldblum, Eran Rotman, Shir Cohen, Einat Brand, Sara Kivity, Mor Saban

**Affiliations:** 1 Maccabi healthcare services, Tel Aviv-Jaffa, 6812509 Israel; 2 Nursing Department, School of Health Professions, Faculty of Medicine, Tel Aviv University, Tel Aviv, Ramat Aviv, 69978 Israel

## Abstract

**Background:**

Conflict profoundly impacts community health and well-being. While post-conflict research exists, little is known about initial effects during active hostilities.

**Objective:**

To assess self-reported changes in health behaviors, distress, and care access within one month of regional warfare onset in a conflict-affected community.

**Methods:**

An online survey was conducted in November 2023 among 501 residents (mean age 40.5 years) of a community where war began October 7th. Measures evaluated physical health, mental health, diet, substance use, sleep, weight changes, and healthcare access before and after the declaration of war.

**Results:**

Relative to pre-war, respondents reported significantly increased rates of tobacco (56%) and alcohol (15%) consumption, worsening sleep quality (63%), elevated distress (18% sought help; 14% needed but didn’t receive it), and postponed medical care (36%). Over a third reported weight changes. Distress was higher among females and those endorsing maladaptive coping.

**Conclusion:**

Within one month, substantial impacts on community psychosocial and behavioral health emerged. Unmet mental health needs and risk-taking behaviors were early indicators of conflict’s health consequences. Continuous monitoring of conflict-affected communities is needed to inform tailored interventions promoting resilience and prevent entrenchment of harms over time.

**Supplementary Information:**

The online version contains supplementary material available at 10.1007/s10900-024-01337-6.

## Background

Armed conflicts wreak immense damage on population health through both physical and psychological impacts [1]. Prolonged exposure to violence and bombardment frequently results in trauma-related disorders like post-traumatic stress disorder, anxiety and depression among conflict-affected communities [1]. Simultaneously, health systems face immense strain responding to surges in injuries from attacks [2–4].

This psychological burden is exacerbated by the immense strain conflicts place on local health systems [5]. Infrastructure is frequently damaged or destroyed by violence. Attacks also force many clinicians and health workers to flee unsafe areas, severely limiting treatment availability for vulnerable groups when needs are greatest [6]. One study highlighted how prolonged exposure to conflict throughout developmentally sensitive periods can biologically program stress responses in the body [5]. This likely explains why those subjected to over a decade of instability and deprivation face such heightened risks of trauma-related disorders [7]. 

Studies on the Syrian conflict highlight its devastating toll on mental health. A systematic review found depression among Syrian refugees and internally displaced persons ranged from 11 to 49%, while anxiety levels ranged from 49 to 55% [8]. More recent research paints an even grimmer picture, with 60% of the Syrian population estimated to be suffering from symptoms consistent with moderate to severe mental disorders [9]. 

A recent quantitative study examined the prevalence of mental health disorders among civilians in Ukraine affected by the ongoing conflict. The researchers found alarming rates of trauma-related issues like depression, anxiety and post-traumatic stress disorder among the study population [10]. 

The 2023 conflict between Israel and Palestinian militant groups Hamas and Islamic Jihad erupted in October 7 [1]. Backed by a barrage of rockets, Hamas militants stormed from the blockaded Gaza Strip into nearby Israeli towns, killing dozens and abducting others in a surprise attack during a major Jewish holiday, [11] triggering renewed rocket fire from Gaza into Israeli communities in the southern area and the warming of the northern district [12]. In response, the Israeli Air Force launched airstrikes targeting militant sites across Gaza [12]. Over 8 weeks, violence escalated rapidly as attacks intensified, jeopardizing civilian lives on both sides of the border. By now, over 1,500 Israelis had been killed and about 7000 are injured [12]. 

While frontline providers worked valiantly to treat injuries and maintain services during crises, accurately assessing long-term health outcomes is critical. Surveying affected populations post-conflict allows gaining a comprehensive understanding of impacts on wellbeing, both immediate and those manifesting over time [13–15]. 

Collecting data on key public health indicators such as conflict-related injuries, prevalence of mental health conditions like post-traumatic stress disorder and depression, access to medical services, and socioeconomic vulnerabilities can help inform a more targeted emergency response by identifying populations most in need of trauma-informed medical, psychological and social support services [2, 15]. Insights from healthcare systems that worked to maintain services during conflict can significantly enhance disaster preparedness and response efforts [5]. 

Following the crisis, a prompt assessment of population health impacts is imperative to effectively respond to public health, physical and mental, needs.

To fulfill this need, researchers at Maccabi Healthcare Services, Israel’s second largest HMO, serving over 2.6 million people, initiated a comprehensive web-based survey in November 2023. This large-scale survey, conducted among a representative sample of the Israeli population, aimed to elucidate and characterize the immediate public health consequences one month after the commencement of hostilities.

As a major service provider that maintained services throughout the bombardment, insights derived from Maccabi’s leading research also had potential to enhance disaster preparedness on a broader scale.

## Methods

### Study Population and Sampling Methods

The study population consisted of Hebrew-speaking citizens aged 20–75. A representative sample of 501 individuals was drawn from this population. To ensure a balanced and unbiased sample the sampling process was monitored with a focus on key demographic factors, including health fund, age, gender and geographic region.

### Ethical Considerations

This study was conducted according to the principles of the Declaration of Helsinki. Approval was received from the University Institutional Review Board prior to commencement of the study (0007724-1). The online survey was distributed through an established research panel (https://www.ipanel.co.il/en/) where participants have already consented beforehand to taking surveys. Careful consideration was given to question wording and order to avoid causing unnecessary distress given the sensitive topic. Confidentiality and anonymity of responses were strictly maintained without collecting any identifying information. Data was securely stored and will only be reported in aggregate form.

### Data Collection

Maccabi Healthcare Services used the internet panel (iPanel) service to distribute the survey. iPanel is an established online research platform administered by the survey company iPanel, for administering surveys among consenting Israeli respondents. The iPanel service recruited a representative 500-person sample of Hebrew-speaking adults aged 20–75 who were health fund members. Participants were screened for eligibility based on the sampling framework.

The online questionnaire was distributed via the iPanel service infrastructure in late November 2023, approximately one month following the initiation of the conflict in October 2023.This timing allowed for assessing short-term public health impacts while events remained relatively recent. The iPanel service platform facilitated efficient data capture from a large, geographically dispersed sample within the study period. Responses were collected anonymously via the iPanel service with robust privacy and confidentiality protocols in place.

### Data Analysis

The collected survey data was analyzed using SPSS software Version 25. Frequencies and percentages were calculated for responses to single-select categorical questions to obtain an overview of patterns in public perceptions and behaviors. Mean scores and standard deviations were also measured for questions using Likert-type rating scales.

Cross-tabulations and chi-square tests were conducted to examine associations between key demographic variables (e.g. gender, age, geographic region) and other factors of interest such as health behaviors, access to care, knowledge levels, and emotional impacts. This helped identify differences in behaviors and perspectives across population subgroups.

## Result

**Fig. 1:**
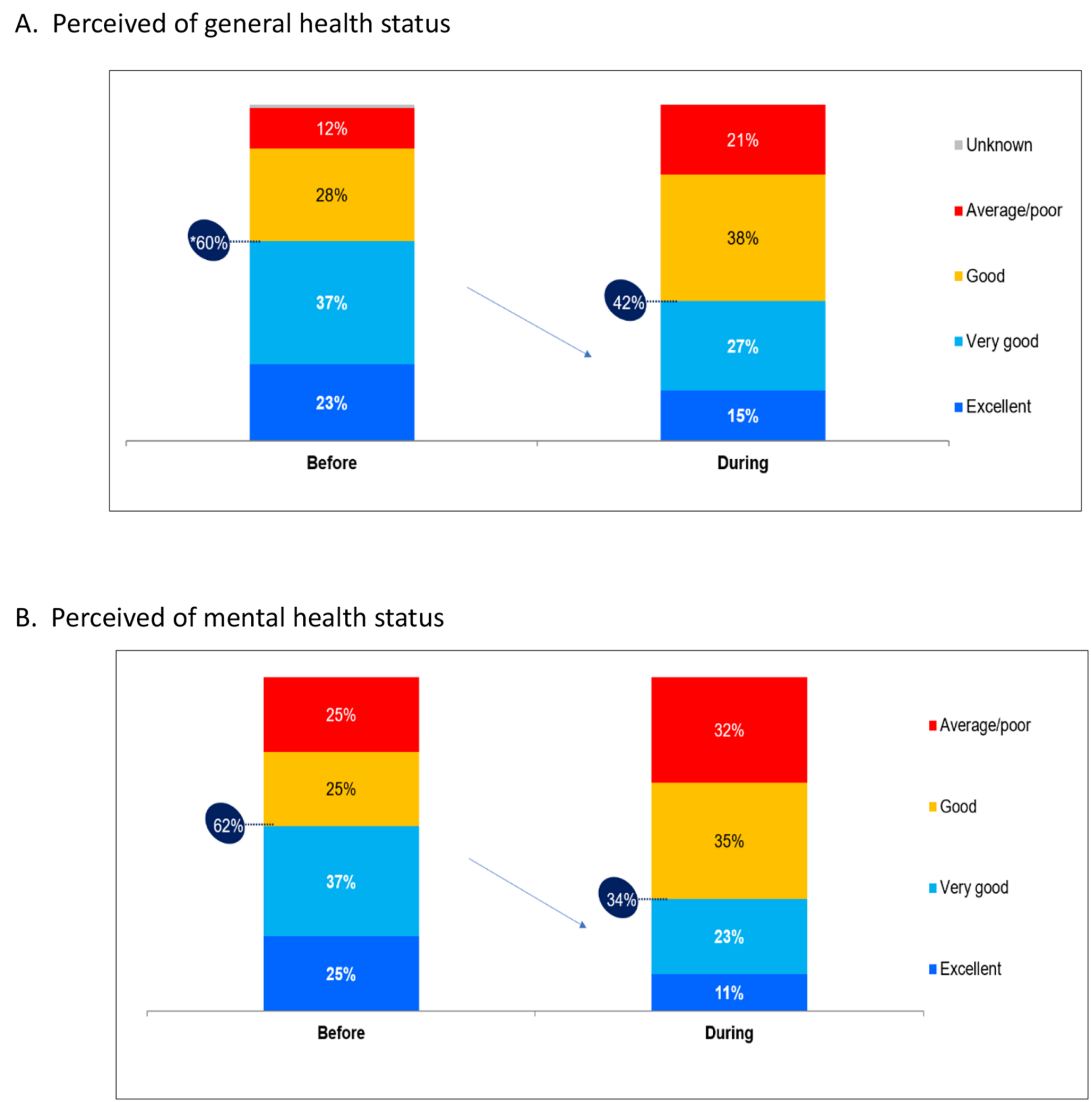
Self-Reported Health Status of Survey Respondents (*N* = 501)

The study included 501 participants. Figure 1 present perceived general health status (Fig. 1A) and Perceived mental health status (Fig. 1B).

There was a decrease in the proportion of people who rate their health status as excellent/very good during the war compared to the period before the war, from 60 to 42%. The proportion of people who perceive their health status as moderate/poor has almost doubled during the war compared to the period before the war. The self-reported health status rating before the war was almost identical to the rating found in a representative nationwide survey conducted in June 2023.

Regrading mental health status, there was a decrease in the proportion of people who rate their mental health status as excellent/very good during the war compared to the period before the war, from 62 to 34%.

According to respondents’ reports, there was no change in the percentage of those taking medication to treat depression or anxiety. However, among those who took medication to treat depression or anxiety before and during the war, 27% increased their dosage or changed medications.

Following the war, every third Israeli reported a change for the worse in their health status, and there was a dramatic decrease in the proportion of Israelis who rated their health as excellent or very good. The proportion of Israelis who perceive their status as moderate or poor has almost doubled during the war and is now 21%, compared to just 12% before the war.

**Fig. 2:**
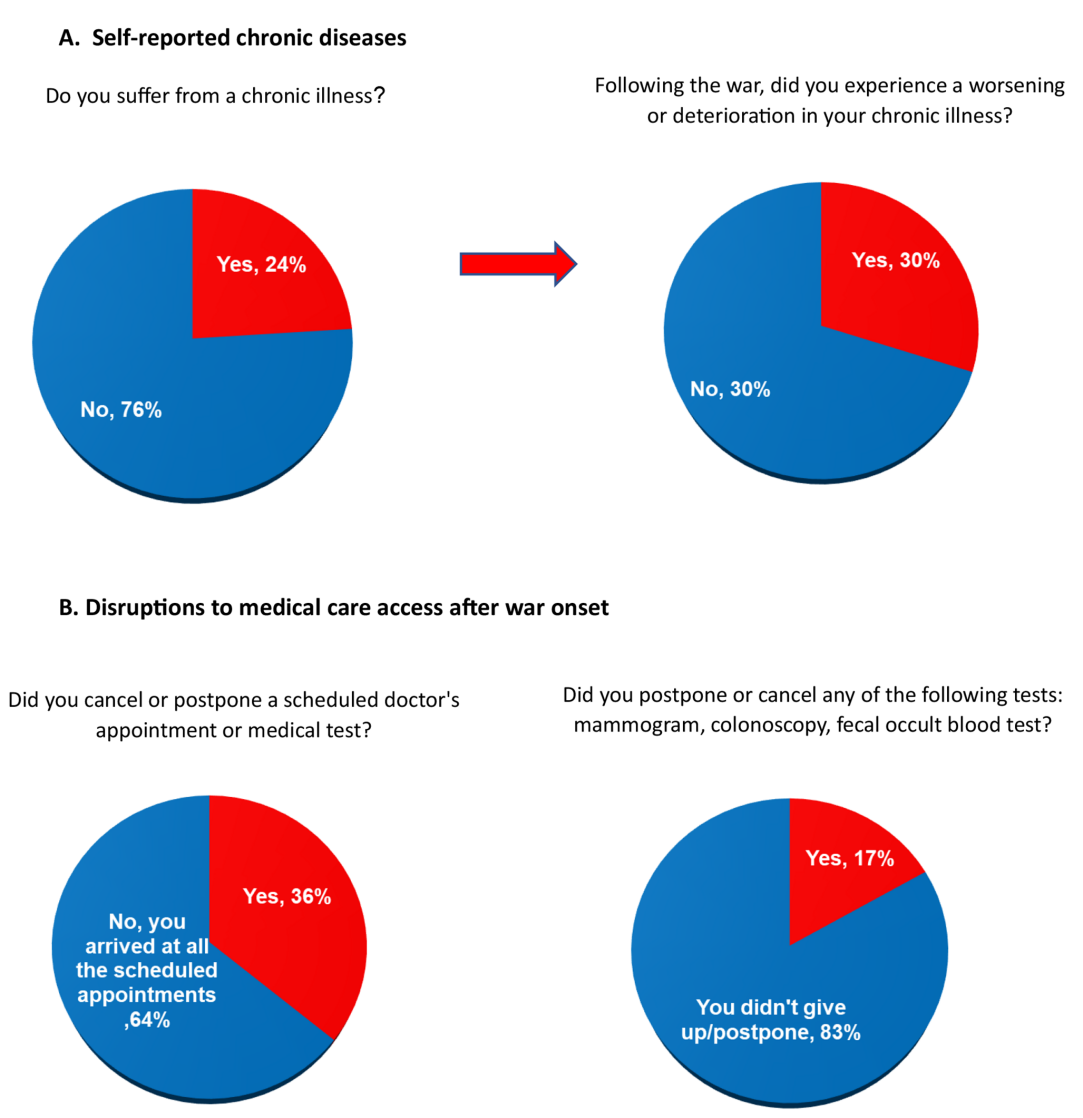
War-Related Effects on Long-Term Health Conditions and Health Appointments (*N* = 501)

The survey data also shows that 30% of Israelis suffering from chronic illnesses, who make up a quarter of the overall population, reported feeling their condition has worsened as a result of the situation (Fig. 2A).

In addition, 36% of respondents who had a scheduled doctor’s appointment or medical test prior to the war reported postponing or canceling the appointment by the patient or the physician. 17% of patients who had scheduled screening test appointments reported postponing or canceling an important screening test, such as a mammogram for early breast cancer detection or colonoscopy for early detection of colorectal cancer. This highlights disruptions to ongoing healthcare and preventative services due to the conflict (Fig. 2B).

**Fig. 3:**
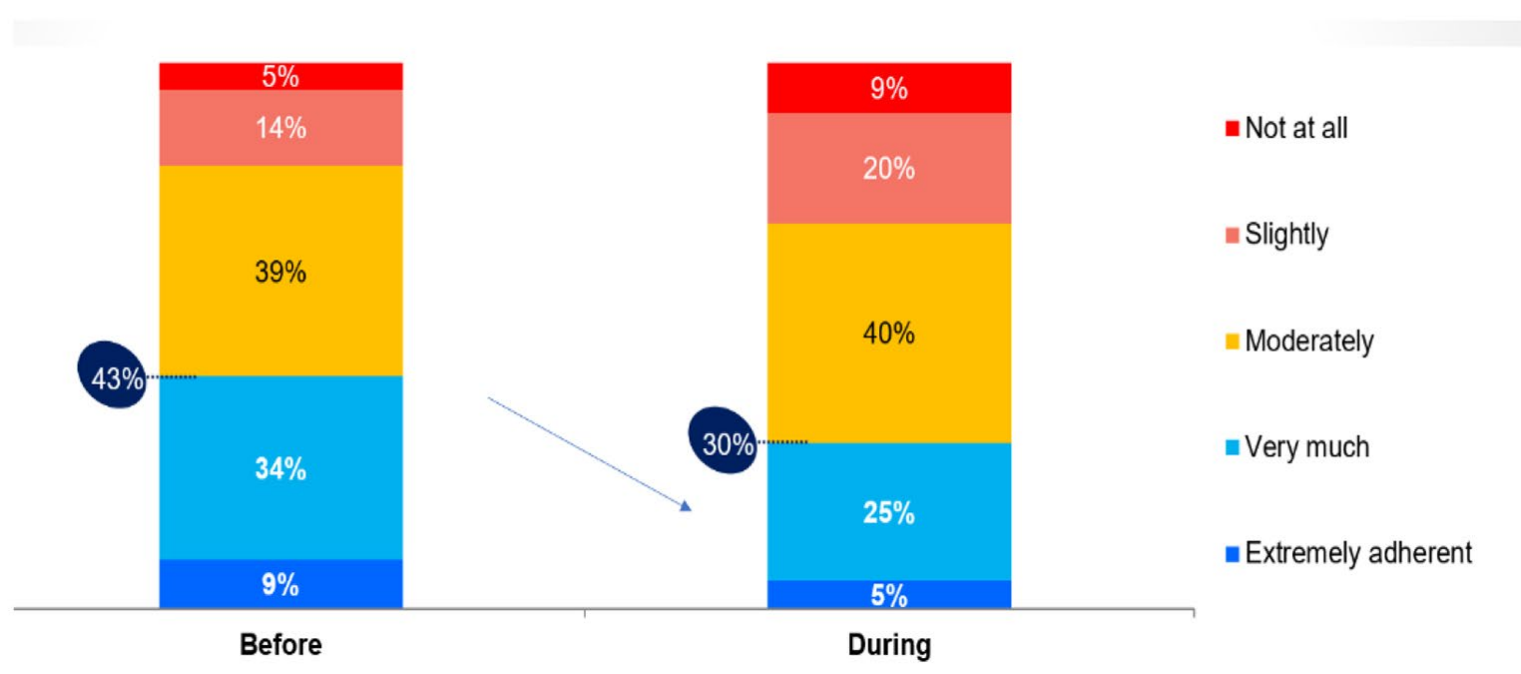
Self-Reported Adherence to Health-Promoting Behaviors (*N* = 501)

The health habits of Israelis also suffered a significant blow due to the outbreak of hostilities. According to survey findings, there was a significant decrease in the proportion of Israelis reporting adherence to a healthy and balanced lifestyle (nutrition, physical activity, smoking, etc.) compared to the pre-war period: from 43% to only 30% (Fig. 3).

About half (47%) of those who exercised and trained regularly before the war testified that they exercise less since the outbreak of the war, and 15% said they stopped training altogether.

**Fig. 4:**
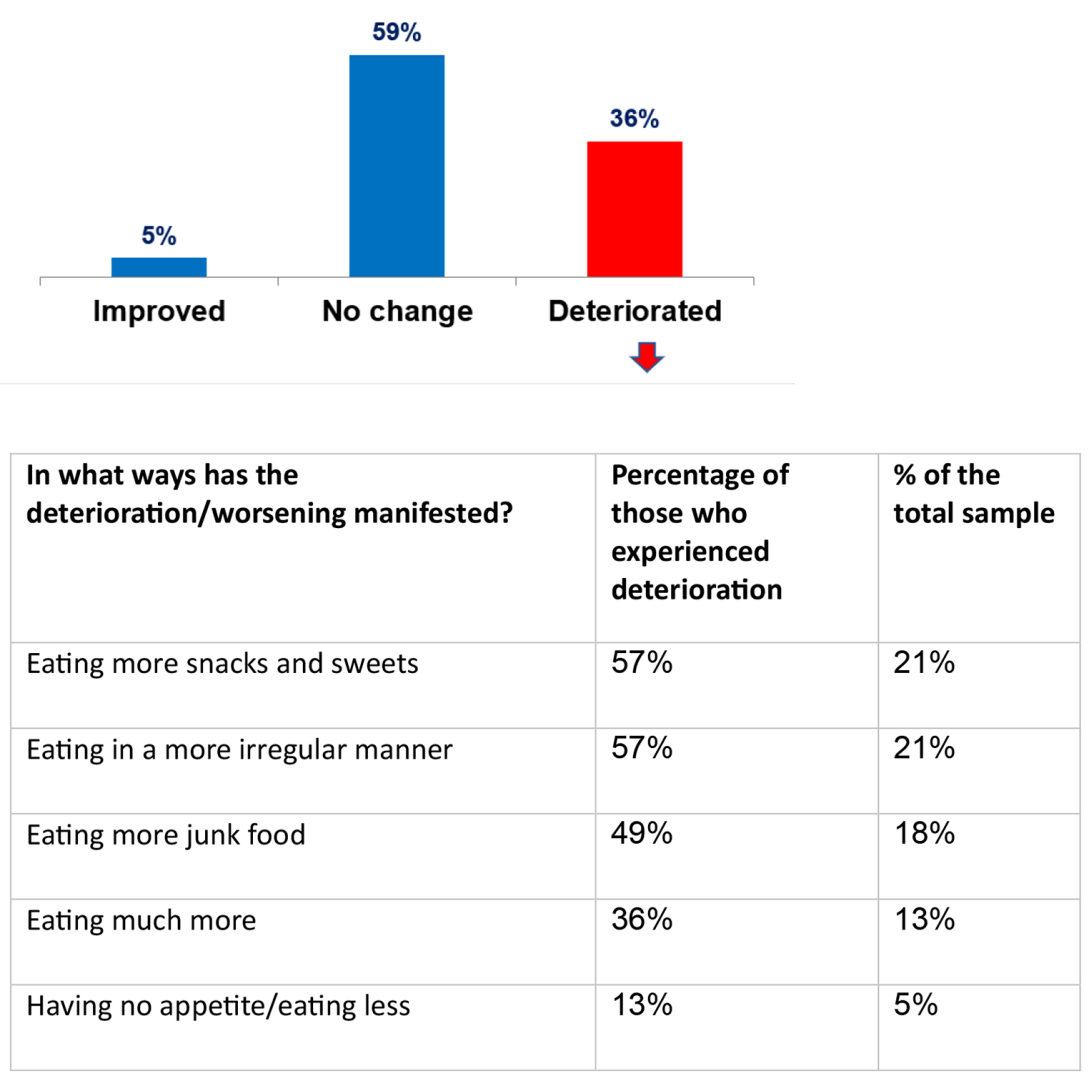
Self-Reported Changes to Eating Patterns Due to Wartime Conditions (*N* = 501)

Of all respondents, 21% reported eating in an irregular manner these days, 21% noted consuming more snacks and sweets during this time, and 18% reported consuming more junk food than usual. At the same time, 13% of respondents said they lost weight as a result of the situation and 36% of respondents reported gaining weight during this period due to a deterioration in their eating habits as a result of the war (Fig. 4).

One of the concerns in the healthcare system is that the current situation could lead to an increase in addictions among the public. The survey findings confirm this fear as about 56% of smokers testified that their cigarette consumption increased during the war period and 10% of those who previously smoked and quit reported returning to smoking. Regarding alcohol consumption, 15% of respondents who normally consume alcohol reported drinking more alcoholic beverages these days.

Moreover, 18% of respondents to the survey reported feeling the need to receive psychological assistance from professionals due to the war. The mental state also directly affects the quality of sleep of Israelis, with 63% of survey respondents noting that they sleep less well these days.

## Discussion

This study provides both important insights and novel findings regarding the direct impacts of war on civilian physical and mental health in Israel after only one month of active conflict. By assessing changes retrospectively from before the war started, the results emerge from the immediate aftermath and thus present a timely perspective.

The results also show a similar decline when compared to pre-war outcomes from June 2023, before the conflict began.

While previous research from prolonged conflicts in Syria [16] and Ukraine [10] documented deteriorations across similar health domains over longer durations, our study revealed that disruptions can manifest rapidly even after a short period of warfare. The high rates of changes observed across lifestyles, behaviors and well-being parameters within the first month align with what might be expected based on other settings, [17] but also demonstrate how acutely conflict can undermine health.

Studies have shown that psychological distress such as depression and anxiety is prevalent amongst civilian populations exposed to violence and instability [18, 19]. The high rates of mental health changes reported in this survey correspond to these observations.

Stress and lack of security are also known to negatively impact sleep quality.

In our study we found a high prevalence of worsening sleep quality, observed in over 60% of respondents, aligns with extensive literature demonstrating that instability and lack of safety undermine peaceful rest [20, 21]. Disrupted circadian rhythms in turn can impair both physical and mental resilience to stress [22]. These findings signal a need for population-level sleep hygiene supports during crises.

The survey findings also indicate that Israelis experienced significant impairments to healthy lifestyle practices as a result of the conflict. Specifically, the survey found a meaningful decline in the proportion of Israelis reporting adherence to balanced, healthy habits related to nutrition, physical activity, smoking etc. - dropping from 43% pre-conflict to just 30%. Nearly half (47%) of regular exercisers said they had reduced their physical activity levels since the fighting began, and 15% reported completely stopping workout routines. This data echoes other research showing that stressful events can undermine self-care and disrupt established healthy behaviors [23, 24]. When faced with crisis situations, adhering to lifestyle protocols may understandably take lower priority over addressing more immediate safety and security needs [23]. 

The changes also fit with patterns of people gravitating towards potentially unhealthy coping strategies like reduced exercise during times of duress. Ensuring residents can maintain health-supporting practices even under pressure likely grows more vital in conflict contexts [25, 26]. 

The survey findings regarding weight changes and eating habits provide further evidence of the impact of conflict on lifestyle practices [25, 27]. The high percentage (36%) reporting weight gain aligns with research showing stress can undermine dietary self-regulation and promote overeating or comfort food consumption. The specific behaviors reported, like irregular eating, snacking more and increased junk food intake, reflect understandably unhealthy coping strategies employed during the unrest. At the same time, some respondents (13%) lost weight, demonstrating a diversity of stress-related impacts.

Ongoing altered lifestyle behaviors during prolonged periods of unrest may compound underlying health vulnerabilities over the long-term, both at an individual level for those with pre-existing conditions, as well as collectively from a public health perspective [28, 29]. Ensuring residents are able to maintain basic health-supporting practices, even under the considerable pressure of conflict contexts, is vital [27].

Screening programs and guidance targeting stress-related coping behaviors could help stabilize routines and mitigate some risks [30]. For example, screening could identify at-risk groups experiencing significant weight changes or disrupted self-care [31]. Brief lifestyle counseling interventions may provide support on stabilizing nutritional habits, recommitting to physical activity routines, and adopting alternative coping strategies to promote wellness despite adversity. This could be especially important for managing chronic diseases or supporting mental health [32]. 

In summary, this study highlighted how the stresses of warfare can pervasively undermine lifestyles, healthcare utilization, and disease management in addition to worsening psychological status. The results lend empirical support to health protection initiatives focused on maintaining services, screening for risks, and bolstering coping resources even under duress. Looking ahead, longitudinal investigation of recovery trajectories as conflict subsides would complement this timely assessment of initial health disturbances. Continued prioritization of population health defense relative to geopolitical unrest remains crucial to civilian welfare.

## Conclusion

This study provides novel insights into the effects of conflict on civilian health behaviors and well-being. Previous research has documented negative impacts, but few quantitative surveys have assessed changes across multiple domains in a representative population sample during ongoing hostilities.

Our results substantiate that armed conflict is associated with widespread deterioration in lifestyle factors, mental health, sleep, and weight patterns among civilians. In particular, increased substance use as an adaptive coping response has received little prior examination but appears common based on our findings.

The implications are that public health initiatives must address an array of health risks stemming from conflict-related stress, rather than focus solely on physical health impacts. Promoting alternative coping strategies and supporting at-risk groups are important to mitigate long-term health consequences.

### Electronic Supplementary Material

Below is the link to the electronic supplementary material.

Supplementary Material 1
